# A Functional Spatial Analysis Platform for Discovery of Immunological Interactions Predictive of Low-Grade to High-Grade Transition of Pancreatic Intraductal Papillary Mucinous Neoplasms

**DOI:** 10.1177/1176935118782880

**Published:** 2018-06-28

**Authors:** Souptik Barua, Luisa Solis, Edwin Roger Parra, Naohiro Uraoka, Mei Jiang, Huamin Wang, Jaime Rodriguez-Canales, Ignacio Wistuba, Anirban Maitra, Subrata Sen, Arvind Rao

**Affiliations:** 1 Department of Electrical and Computer Engineering, Rice University, Houston, TX, USA; 2 Department of Computational Medicine and Bioinformatics, University of Michigan, Ann Arbor, MI, USA; 3 Department of Translational Molecular Pathology, The University of Texas MD Anderson Cancer Center, Houston, TX, USA; 4 Department of Anatomical Pathology, The University of Texas MD Anderson Cancer Center, Houston, TX, USA; 5 Department of Radiation Oncology, University of Michigan, Ann Arbor, MI, USA

**Keywords:** IPMN, multiplex immunofluorescent (mIF) imaging, spatial statistics, functional data analysis, machine learning

## Abstract

Intraductal papillary mucinous neoplasms (IPMNs), critical precursors of the devastating tumor pancreatic ductal adenocarcinoma (PDAC), are poorly understood in the pancreatic cancer community. Researchers have shown that IPMN patients with high-grade dysplasia have a greater risk of subsequent development of PDAC in the remnant pancreas than do patients with low-grade dysplasia. In this study, we built a computational prediction model that encapsulates the spatial cellular interactions in IPMNs that play key roles in the transformation of low-grade IPMN cysts to high-grade cysts en route to PDAC. Using multiplex immunofluorescent images of IPMN cysts, we adopted algorithms from spatial statistics and functional data analysis to create metrics that summarize the spatial interactions in IPMNs. We showed that an ensemble of models learned using these spatial metrics can robustly predict, with high accuracy, (1) the dysplasia grade (low vs high grade) and (2) the risk of a low-grade cyst progressing to a high-grade cyst. We obtained high classification accuracies on both tasks, with areas under the curve of 0.81 (95% confidence interval: 0.71-0.9) for task 1 and 0.81 (95% confidence interval: 0.7-0.94) for task 2. *To the best of our knowledge, this is the first application of an ensemble machine learning approach for discovering critical cellular spatial interactions in IPMNs using imaging data*. We envision that our work can be used as a risk assessment tool for patients diagnosed with IPMNs and facilitate greater understanding and investigation of the cellular interactions that cause transition of IPMNs to PDAC.

## Introduction

Intraductal papillary mucinous neoplasms (IPMNs) are cystic lesions that are potential forerunners of pancreatic ductal adenocarcinoma (PDAC).^1^ Histologically, IPMNs are characterized by mucin-filled cysts that grow in the ductal system of the pancreas (main duct and/or branches) and are covered by tall, columnar papillary neoplastic epithelium with variable mucin secretion and various degrees of dysplasia; the sub-epithelial stroma lacks the ovarian-type stroma found in mucinous cystic neoplasms.^2^ Noninvasive IPMNs are classified using a 2-tier system: IPMNs with low-grade dysplasia (low to intermediate dysplasia) and IPMNs with high-grade dysplasia based on the highest degree of cytological and architectural atypia.^3^ Compared with noninvasive IPMNs, IPMNs with associated PDAC have a worse prognosis, with 5-year overall survival rates ranging from 24% to 40%.^4,5^

Pancreatic adenocarcinoma has been shown to have a highly immune-suppressive microenvironment with low densities of intratumor T-cell infiltration and cytotoxic T cells; several studies have shown that this immunosuppression is complex and some of the key players identified are the presence of tumor-associated macrophages and the expression of immune checkpoint biomarkers, such as programmed cell death protein 1 (PD-1) in T cells (antigen-experienced T cells) and programmed death-ligand 1 (PD-L1) in tumor cells as well as in immune cells especially in macrophages.^6–8^ We have selected these relevant biomarkers, and spatial interactions between them, to determine the immune landscape of IPMN lesions and its association with progression.

Currently, broad morphological features serve as the only guidelines available to decide whether a patient diagnosed with an IPMN should undergo a surgical intervention.^9,10^ Resection is recommended for patients with either high-grade dysplasia or an IPMN that has progressed to PDAC, which necessitates accurate grading of dysplasia.^11^ Presently, gold standard prognosticators for assessing cancer risk in patients with IPMNs are lacking.^12^ However, researchers have shown that patients with high-grade dysplasia tend to be at greater risk for subsequent development of PDAC after resection than are patients with low-grade dysplasia.^13^ Also, authors recently reported that the role of the tumor microenvironment, specifically, the spatial interactions between tumor cells and various immune cells, is important in predicting survival in PDAC^14^ and breast cancer.^15,16^

Given the previous findings described above, our goal in this study was to analyze the cell-cell spatial interactions in IPMNs that correlate with dysplasia grade and predict the risk of transition from low-grade to high-grade cysts. In this study, we adopt ideas from spatial statistics (the G-function^17^) and functional analysis^18^ to quantify the spatial tumor environment as observed using multiplex immunofluorescent (mIF) images of IPMN cysts. Researchers have previously used the G-function in ecology to quantify distance-based relationships between predators and their prey^19,20^ and used functional analysis to efficiently encode information in structured data, such as time series data (temporal structure)^21,22^ and images (spatial structure).^23,24^ Very recently, authors have shown that G-functions representing spatial interactions between tumor cells and regulatory T cells predict poor overall survival in patients with non–small-cell lung cancer (NSCLC).^25^ They compute a single metric (“area under curve”) from the G-function, thus omitting a significant amount of the rich information contained in the G-function. As an extension to this approach, we derived multiple spatial metrics from the G-function to build ensemble learning models that perform very well in (1) classification of IPMN dysplasia grade and (2) predicting risk of progression from low-grade to high-grade IPMN cysts by examining mIF images of low-grade cysts alone. We observed high classification areas under the curve (AUCs) of 0.81 (95% confidence interval [CI]: 0.71-0.9) for task 1 and 0.81 (95% CI: 0.7-0.94) for task 2.

## Materials and Methods

### Case selection, staining, and analysis

Formalin-fixed, paraffin-embedded (FFPE) histological IPMN sections were prospectively taken from surgically resected specimens that were obtained from 31 patients who had undergone surgery with curative intent at The University of Texas MD Anderson Cancer Center. Of these patients, 12 had IPMNs with low-grade dysplasia, 17 had IPMNs with high-grade dysplasia, and 2 had invasive carcinoma. In all, 16 of the high-grade IPMN patients also had low-grade dysplasia. Because our subsequent prediction tasks related to only low-grade and high-grade dysplasia, the 2 patients with invasive carcinoma were omitted, leaving a total of 29 patients. The cases were reviewed by 2 pathologists who are experts in assessment of pancreatic lesions (H.W. and A.M.) and classified according to guidelines presented by Raimondo et al.^4^

Manual mIF staining as described previously^26^ was performed with 4-µm-thick sequential histological tumor sections obtained from a representative FFPE tumor block using the Opal 7-Color Kit (PerkinElmer, Waltham, MA, USA) and scanning using a Vectra multispectral microscope (PerkinElmer). The IF markers used were grouped into a 6-antibody panel, consisting of pancytokeratin AE1/AE3 (epithelial cell positivity; dilution, 1:300; Dako, Carpinteria, CA, USA), programmed death-ligand 1 (PD-L1; clone E1L3N; dilution, 1:100; Cell Signaling Technology, Beverly, MA, USA), CD3 (T-cell lymphocytes; dilution, 1:100; Dako), CD8 (cytotoxic T cells; clone C8/144B; dilution, 1:20; Thermo Fisher Scientific, Waltham, MA, USA), programmed cell death protein 1 (PD-1; clone EPR4877-2; dilution, 1:250; Abcam, Cambridge, MA, USA), and CD68 (macrophages; clone PG-M1; dilution, 1:450; Dako). The methodology of the multiplex immunofluorescence staining has been reported before,^26^ specifically, the staining of the antibodies was performed consecutively using the Opal 7 multiplexed assay. The validation of the assay included the optimization of the antibodies using bright field immunohistochemistry, assessment of the uniplex immunofluorescence, and optimization of the multiplex immunofluorescence staining to ensure that the antibodies stain correctly. The use of the Vectra multispectral microscope (PerkinElmer) and the inForm 2.1.0 software program (PerkinElmer) allows a robust identification of the fluorescence signal and the co-localization of the biomarkers.

**Figure 1.**
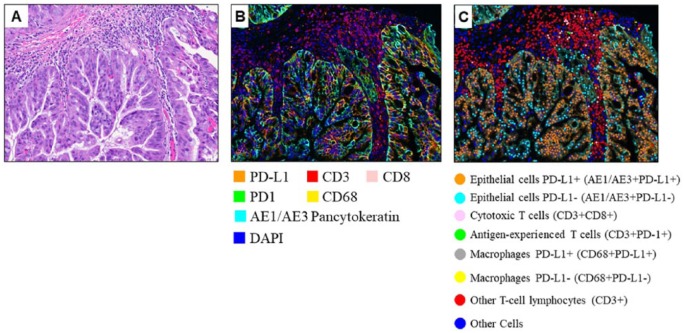
The mIF images of IPMN. (A) Hematoxylin-eosin, (B) multiplexed immunofluorescence, and (C) phenotyped mIF images of an IPMN with high-grade dysplasia. The cell types of interest are highlighted in C using different colors as indicated. IPMN indicates intraductal papillary mucinous neoplasm; mIF, multiplex immunofluorescent. The hematoxylin-eosin image (A) was acquired at 20x magnification.

A total of 219 mIF IPMN images containing the different markers were analyzed using the inForm 2.1.0 software program (PerkinElmer). For every patient, about 5 images (total physical dimensions: 2.1 mm × 1.6 mm, with a 1.57-µm/pixel resolution) were captured for each low-grade and high-grade region. Example mIF images of IPMN with high-grade dysplasia are shown in Figure 1. In the images, individual cells were identified using nuclear (4′,6-diamidino-2-phenylindole) staining, and a phenotyping pattern recognition learning algorithm tool was used to characterize co-localization of the various cell populations (Table 1) and create a comprehensive cell-by-cell identification report using the antibody markers in the epithelial and stromal compartments of the lesions. The individual cell report created by inForm, including the spatial location (X- and Y-coordinates) of each cell, was finally processed using the Spotfire software program (TIBCO Software, Palo Alto, CA, USA; PerkinElmer) to create a final data report for every image. The pairwise combinations whose spatial interactions we investigated are listed in Table 2.

**Table 1.** Cell types of interest: the cell types whose spatial locations were quantified in mIF images.

Cell type
Epithelial cells (AE1/AE3+)
Epithelial cells PD-L1+ (AE1/AE3+PD-L1+)
T-cell lymphocytes (CD3+)
Antigen-experienced T cells (CD3+PD-1+)
Cytotoxic T cells (CD3+CD8+)
All macrophages (CD68+)
Macrophages PD-L1+ (CD68+PD-L1+)

**Table 2.** Spatial interactions of interest: the various pairwise combinations of cell types interrogated in our spatial analysis framework.

Spatial interactions of interest
1. All epithelial cells vs all T-cell lymphocytes
2. All epithelial cells vs cytotoxic T cells
3. All epithelial cells vs antigen-experienced T cells
4. Epithelial cells (PD-L1+) vs all T-cell lymphocytes
5. Epithelial cells (PD-L1+) vs cytotoxic T cells
6. Epithelial cells (PD-L1+) vs antigen-experienced T cells
7. All macrophages vs all T-cell lymphocytes
8. All macrophages vs cytotoxic T cells
9. All macrophages vs antigen-experienced T cells
10. Macrophages (PD-L1+) vs all T-cell lymphocytes
11. Macrophages (PD-L1+) vs cytotoxic T cells
12. Macrophages (PD-L1+) vs antigen-experienced T cells

### Functional spatial analysis

#### G-function: basic idea

**Figure 2.**
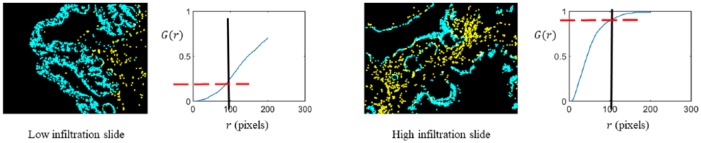
G-function explanation: we provide here a visual description of our G-function analysis. Epithelial cells are shown in blue, and cytotoxic T cells are shown in yellow. The G-function was compared for 2 scenarios: low cytotoxic T-cell infiltration (left) and high cytotoxic T-cell infiltration (right). In the low infiltration image, the G-function rises slowly, going upto a value of about only 0.25 at r=100pixels. However, the G-function rises rapidly in the case of the high infiltration image, reaching a value of about 0.9 at r=100pixels (1 pixel = 1.57 microns). The G-function is a signature of the nature and extent of infiltration of cells of one type into the other.

The spatial G-function is used to quantify infiltration of cells of one type into another.^17^ Specifically, the G-function computed a nearest neighbor distribution function for cells of type “*j*” with respect to cells of type “*i*.” As shown in Figure 2, the G-function computed as a function of distance “*r*” informed us of the probability of a cell of type “*i*” having at least one cell of type “*j*” within a distance *r* from it. Different levels of infiltration clearly had signature G(r) curves.

#### G-function: summary metrics

**Figure 3.**
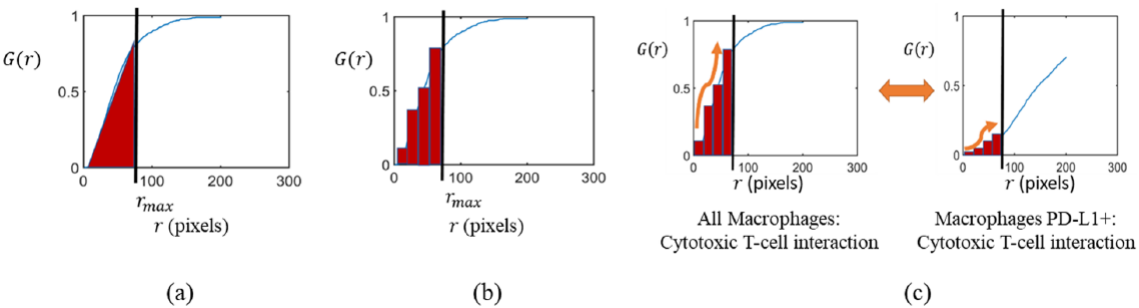
G-function summary metrics. (a) Simple AUC. The G-function curve is summarized using one number, the AUC, for 0 ≤ *r* ≤ *r*_*max*_. The simple AUC, while compact, lacks information about the shape of the curve. (b) K-bins AUC. The range of *r* over which the G-function is computed is split into K-bins $\left(0\leq r_{1}\leq r_{2}\leq \ldots \leq r_{K-1}\leq r_{\max}\right)$ and the area under the G-function is computed for each bin. Thus, each curve is represented by K numbers. (c) K-bins MFPCA. To analyze P spatial interactions, the number of bins becomes P*K. To account for interbin relationships for a given spatial interaction and the correlations among various spatial interactions, such as the pair of interactions shown, an MFPCA approach is used. The information contained in the P*K numbers required for the K-bins metric can be almost entirely preserved using only a few “k” numbers, which are the principal component scores generated by MFPCA. AUC indicates area under the curve; MFPCA, multivariate functional principal component analysis.

To design efficient machine learning schemes, the entire G-function was summarized by formulating the following 3 different metrics as shown in Figure 3:

1. Simple AUC: a clinically meaningful maximum value of the cell-to-cell distance *r*, which we call $r_{max}$, was chosen, and the area under the G-function for 0 ≤ *r* ≤ *r*_*max*_ was computed as shown in Figure 3(a). This simple AUC metric, used recently in quantifying spatial interactions in NSCLC,^25^ is thus one simple number; however, the shape information of the curve is ignored.
2. K-bins AUC: the G-function was partitioned into K-bins, and AUCs for each bin were computed separately as shown in Figure 3(b). This metric better preserves shape information than the simple AUC while still being a compact representation.
3. K-bins multivariate functional principal component analysis (MFPCA): our intention is to query spatial interactions for the P pairwise combinations listed in Table 2 (P = 12 for the current study). A naïve way of representing each mIF image would be to simply concatenate the K-length metric from step 2 for each of the P interactions. However, because these P interactions are correlated (for example, all macrophages vs cytotoxic T-cell and macrophages PD-L1+ vs cytotoxic T-cell interactions are expected to have similar G-functions as seen in Figure 3(c)), the (P*K) number of AUC values from the previous step can be more efficiently summarized using MFPCA.^18,27^ The MFPCA algorithm proceeds in two steps: first, an FPCA step that reduces the correlations across bins, and second, a multivariate eigen-value decomposition step that decreases correlations across multiple interactions. The FPCA step computes principal component scores for each of the P G-functions, while the multivariate eigen-value decomposition condenses the information in these scores to even fewer components. The algorithm is designed such that these top components are generated using only a few selected spatial interactions, thus providing insight into which interactions are critical. Overall, MFPCA reduces the length of the metric from (P*K) to a smaller number k while explaining 95% of the variation in the (P*K) AUC values.

The G-function computations were performed using the R language “spatstat” package.^28^ The simple and K-bins AUC metrics were calculated using MATLAB (MathWorks, Natick, MA, USA). The K-bins MFPCA metric was computed using the “MFPCA” package in R.^29^ A random forest prediction model for each of the different metrics was built using the “randomForest” package in R.^30^

## Results

The 219 mIF images we used in this study were composed of 129 low-grade and 90 high-grade IPMN images. Of the low-grade images, 59 were from patients with low-grade cysts only, whereas 70 were from patients who also had high-grade cysts. We compared our spatial interaction metrics with 2 other metrics: a simple count of cells exhibiting each phenotype listed in Table 1 and the Morisita-Horn index,^31^ a spatial co-localization metric that has been shown to be prognostic for breast cancer.^16^

We built a random forest prediction model using 500 decision trees.^32^ The random forest method has been shown to be robust to overfitting and among the most effective of the widely used classifiers. We then cross-validated our prediction results using a leave-one-out approach.^33^ We built ensemble learning models by combining the predictions from the different spatial interaction models and a counts-only model, as these 2 models capture complementary information about the data (spatial proximity between cells and cell abundance, respectively). For the G-function AUC computations, we experimented with multiple values of the parameter $r_{max}$ between 10 *µm* (6 pixels) and 100 *µm* (60 pixels) so as to capture spatial interactions over a wide range of distances. We observed that the best performance for task 1 was obtained by setting r_max_ = 32 µm (20 pixels) for the G-function AUC model, K = 7 for the G-function K-bins model, and k = 7 for the G-function K-bins MFPCA model. For task 2, we obtained the best results by setting r_max_ = 24 µm (15 pixels) for the G-function AUC model, K = 14 for the G-function K-bins model, and k = 8 for the G-function K-bins MFPCA model.

### Classification of low-grade vs high-grade dysplasia

**Figure 4.**
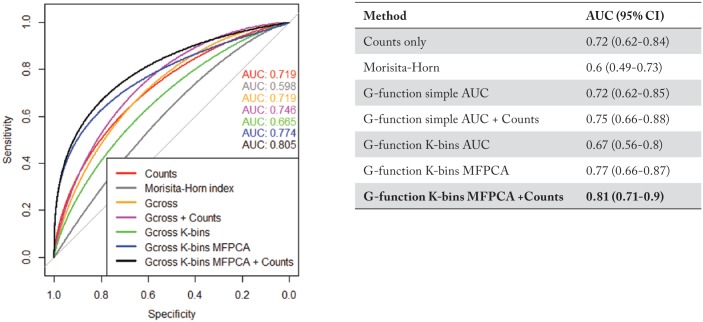
Low-grade vs high-grade classification: performance analysis of various methods used to classify low- and high-grade dysplasia in IPMN patients. The best classifier was obtained using an ensemble of the G-function K-bins MFPCA model and a counts-only model, with an AUC of 0.81 (95% CI: 0.71-0.9). (Note: The term Gcross in the figure legend denotes the G-function simple AUC metric, while Gcross K-bins denotes G-function K-bins AUC metric). AUC indicates area under the curve; IPMN, intraductal papillary mucinous neoplasm; MFPCA, multivariate functional principal component analysis.

We show the receiver operating characteristic (ROC) curves, classification AUCs, and respective CIs for each classification method in Figure 4. The ROC curves are all significantly better (*p* < 0.05) than an ROC curve resulting from random chance (AUC 0.5), which is the 45° straight line in Figure 4. We observed that an ensemble model built using the predictions from the G-function K-bins MFPCA model and counts-only model had the best classification performance, with an AUC of 0.81 (95% CI: 0.71-0.9).

### Classification of low-grade dysplasia mIF images in patients with only low-grade cysts vs. those with concurrent low- and high-grade cysts

**Figure 5.**
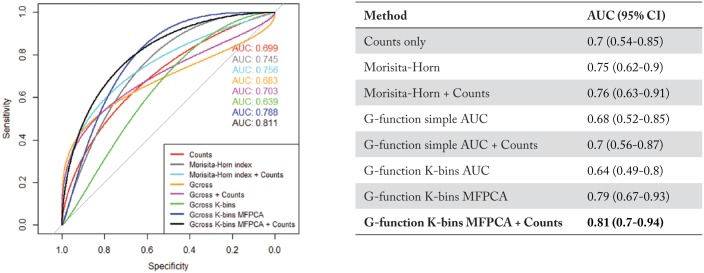
Low-grade only vs mixed low-grade/high-grade classification task: Performance analysis of various methods used to classify low-grade mIF images for IPMN patients with only low-grade dysplasia vs patients with both low- and high-grade dysplasia. The best approach was an ensemble of the G-function K-bins MFPCA model and a simple counts-only model, with an AUC of 0.81 (95% CI: 0.7-0.94). (Note: The term Gcross in the figure legend denotes the G-function simple AUC metric, while Gcross K-bins denotes G-function K-bins AUC metric). AUC indicates area under the curve; CI, confidence interval; IPMN, intraductal papillary mucinous neoplasm; mIF, multiplex immunofluorescent.

The ROC curves, classification AUCs, and respective CIs for each of the classification methods are shown in Figure 5. Just as the previous task, all the approaches performed significantly better (p<0.05) than random chance. Once again, we obtained the best performance using an ensemble classifier built by combining the predictions from the G-function K-bins MFPCA model and a counts-only model. It achieved a classification AUC of 0.81 (95% CI: 0.7-0.94).

### Important spatial interactions

The top 3 spatial interactions ranked according to the mean decrease in classification accuracy (%) criterion are listed in Table 3. This criterion represents the amount by which the classification performance of our learned ensemble model drops if a particular spatial interaction is removed. We noticed that the spatial interaction of epithelial cells with T-cell lymphocytes was by far the most important correlate of dysplasia grade in IPMNs, as removing it would lower classification performance by 17%. Interactions between Macrophages PD-L1+ and all T-cell lymphocytes and between epithelial cells and cytotoxic T cells, were also quite significant (7.9% and 7% decrease in accuracy). However, the spatial interaction between Macrophages PD-L1+ and cytotoxic T cells, epithelial cells and all T-cell lymphocytes, and between Macrophages PD-L1+ and cytotoxic T cells were identified by our models as the most important interactions in predicting the likelihood of a concurrent high-grade cyst in a patient with a low-grade cyst (9%, 7.5%, and 5% decrease in accuracy, respectively).

**Table 3.** Important spatial interactions: listed here are the 3 most important spatial interactions for the 2 classification tasks.

Low-grade vs high-grade		Low-grade only vs mixed low-grade/high-grade	
Spatial interaction	Mean decrease in classification accuracy (%)	Spatial interaction	Mean decrease in classification accuracy (%)
Epithelial cells and All T-cell lymphocytes	17.1	All macrophages and cytotoxic T cells	9.0
Macrophages PD-L1+ and all T-cell lymphocytes	7.9	Epithelial cells and all T-cell lymphocytes	7.5
Epithelial cells and cytotoxic T cells	7.0	Macrophages PD-L1+ and cytotoxic T cells	4.9

The interactions are ordered according to mean decrease in classification accuracy (%), which quantifies the drop in classification performance if the effect of that spatial interaction is removed from the analysis.

## Discussion

A comprehensive understanding of the tumor immune environment is essential to understand the causes of progression of IPMNs, particularly in the era of immunotherapy. In this work, we quantified the precise spatial relationships among various cells in IPMNs, rather than performing simple cell counts, as an essential step in gaining insight into why low-grade IPMN cysts progress to high-grade ones. To that end, we adopted the G-function, used historically to study predator-prey interactions in ecology, as a quantitative descriptor of the spatial distribution of specific cells of interest. We summarized the G-function information in 3 ways. The first, called the simple AUC, is simply computation of the area under the G-function curve upto a given distance of interest. The second, called the K-bins AUC, splits the G-function curve into K segments and then computes their corresponding K AUCs. This method can capture the cell-to-cell distances at which key spatial interactions happen more precisely than the simple AUC. Because we investigate several (say N) correlated spatial interactions, we encoded the G-function in a third way, called the K-bins MFPCA, which efficiently represents the large, correlated set of N*K numbers with only a small set of k uncorrelated numbers. Each patient is thus represented by N, N*K, and k numbers, respectively, for the 3 G-function summary metrics we computed. We then used them as inputs in a random forest model for 2 tasks of interest: (1) identifying low-grade cysts and high-grade IPMN cysts and (2) predicting the risk of a low-grade cyst progressing to a high-grade cyst. We also compared the performance of our G-function metrics with a simple cell counts-only model and another spatial co-localization metric called the Morisita-Horn index. We observed that a model that combines the information from the G-function K-bins MFPCA model and the counts-only model achieved the best prediction performance in task 1, with an AUC of 0.81 (95% CI: 0.71-0.9). In task 2, we similarly saw that a model that combines information from the G-function K-bins MFPCA model and the counts-only model achieved the highest prediction performance, with an AUC of 0.81 (95% CI: 0.7-0.94).

The mIF imaging methods that can facilitate the simultaneous identification of proteins can increase our ability to study individual cells and their spatial distribution in several types of tissue. Although cell counts have been traditionally used to understand immune context,^34^ an added incorporation of spatial measures of infiltration demonstrates an improved performance in these classification tasks. By combining the capabilities of image analysis software and current spatial analysis algorithms, our mIF-based functional spatial analysis platform can improve the performance of high-throughput immune profiling and spatial distribution assays in studying premalignant progressive tumor specimens. The identification of prognostic spatial patterns of cell populations in the microenvironment of premalignant tissue can help increase understanding of disease progression, identifying new treatments, and personalizing treatment and prevention strategies.

This study demonstrated that the spatial proximities between epithelial cells and T-cell lymphocytes, specifically, cytotoxic T cells, are predictive of the dysplasia grade for an IPMN cyst. The dissimilar spatial landscapes of low-grade and high-grade cysts indicate that in high-grade cysts, immune-suppressive mechanisms in epithelial IPMN cells lead to suppression of cytotoxic activity and may lead to progression to PDAC. The finding that the spatial distribution of both Macrophages PD-L1+ and all macrophages with respect to cytotoxic T-cell lymphocytes in low-grade IPMN can predict the presence of simultaneous low- and high-grade dysplasia, which indicates that macrophages may suppress cytotoxic T-cell activity early in the development of PDAC. This agrees with in vivo studies of pancreatic adenocarcinoma mouse models suggesting that tumor-associated macrophages suppress cytotoxic T-cell activity to regulate tumor development.^35–37^ The role of macrophages in development of preneoplastic pancreatic lesions such as IPMNs has not been well established, so our findings warrant further investigation. The results suggest that overall, interactions between epithelial cells and lymphocytes, and between macrophages and lymphocytes, are most relevant in these tasks. Thus, there is a strong similarity in the nature of these top-ranking spatial interactions for the 2 tasks. The reasons behind the important interactions being not identical need to be studied on a larger cohort.

This study has limitations that must be addressed in the future. First, we expect our ensemble learning model to perform more robustly with sample sizes greater than those used in this study; thus, follow-up validation studies on larger cohorts are essential. Second, we acquired the patient cohort under carefully controlled sample processing, staining, and imaging protocols; hence, observing the generalizability of these derived prediction models to images acquired under different conditions, for example, across different hospitals, would be important to establish the generalizability of our approach. Third, we only investigated a small set of 12 potentially important spatial interactions of interest. However, analyzing other spatial interactions, potentially with new markers such as granzyme B and CD45RO, is possible, which can shed more light on the poorly understood progression of IPMNs. Fourth, from the clinical standpoint, our study did not include follow-up data or have a sample size sufficient to fully characterize the immune profiles of patients with invasive pancreatic adenocarcinoma, who have markedly worse outcomes than patients diagnosed with IPMN. Finally, it is important to find out whether our proposed G-function metrics are associated with IPMN progression independently of relevant clinical and radiologic predictors. We aim to investigate this in an upcoming study.

## Conclusions

We developed a prediction model based on spatial statistics and functional data analysis to distinguish the spatial microenvironments of low- and high-grade dysplasia in IPMNs. Our model identified the spatial interactions in IPMNs that can predict grade of dysplasia and the likelihood of a low-grade dysplasia transitioning to a high-grade one. Based on a spatial statistical algorithm, the G-function, we derived multiple metrics to summarize the extent of infiltration between multiple cell types of interest as seen in mIF images. For classification of low-grade vs high-grade dysplasia, we achieved the best AUC of 0.81 by combining a simple counts-only model with a model built by summarizing the G-function using MFPCA. To distinguish low-grade cysts belonging to a patient with only low-grade cysts, as opposed to a patient with concurrent low- and high-grade cysts, we obtained the best AUC of 0.81 using an ensemble of a counts-only model with a model that summarizes the G-function using MFPCA. The G-function K-bins MFPCA model is implemented using the fast and open-source R software package MFPCA^29^ and so consequently can be introduced in a clinical setting with minimal cost and effort. Our prediction model is an objective risk assessment tool for patients diagnosed with IPMNs and can potentially be used as a guideline for designing treatment. Furthermore, the key spatial interactions identified in our study can be studied and characterized further from a tumor microenvironment standpoint.
